# A large thigh mass: a blood clot or a rare skeletal muscle metastasis from renal cell carcinoma

**DOI:** 10.1186/2193-1801-2-399

**Published:** 2013-08-23

**Authors:** Vipin Lohiya, Sheela Lohiya, Kevin Windsor

**Affiliations:** Internal medicine, Baptist Health System, 800, Montclair Rd, Birmingham, 35203 Alabama USA; Birmingham Oncology Associates, Trinity Medical center, Birmingham, Alabama USA

**Keywords:** Renal cell carcinoma, Skeletal muscle metastasis, FDG-PET/CT

## Abstract

**Background:**

Renal cell carcinoma (RCC) is a tumor known for its unusual presentations and high rate of metastasis. Metastasis to lung, liver, bone and brain are common, but to skeletal muscle(SM) is very rare. Because only 11% of the RCC metastases to SM present after 10 years of initial presentation, there is no general consensus for its annual surveillance.

**Methods:**

We report a case of a 58 year old male with a history of RCC, initially diagnosed 11 years ago, who presented with a large SM mass. A large mass measuring more than 25 cm was located in left posterior thigh and was present for more than a year. It initially was diagnosed as a large blood clot and was treated with warfarin for more than 6 months. Clinical work up including FDG-PET/CT and MRI raised the possibility of a tumor, but a negative biopsy made the diagnosis uncertain. Because of high suspicion for a tumor, patient underwent a complete resection of the mass.

**Results:**

The resected mass measuring 28 × 18 × 7 cm was detailed as the largest skeletal muscle metastasis from RCC ever reported.

**Conclusion:**

This case emphasizes the importance of maintaining a high suspicion for metastasis even in less common metastatic sites mainly in patients with a history of RCC. It also highlights the importance of annual surveillance for metastasis in patients with RCC even after 10 years of initial presentation using FDG-PET/CT.

## Introduction

Skeletal muscle is a rare site of metastasis accounting for <1% of metastasis.(Pompo et al. 2008; Camnasio et al. 2010; Gözen et al. 2009) Lungs and GI tumors are common, but rarely RCC, head and neck carcinomas can also present with skeletal metastasis. (Pompo et al. 2008; Gözen et al. 2009) In recent large autopsy series, it was found that less than <1% of the RCCs metastasized to skeletal muscle. (Pompo et al. 2008; Ali et al. 2011; Camnasio et al. 2010). From recent review of English literature, only 35 cases of skeletal muscle metastasis from RCC have been reported (Sountoulides et al. 2011) and of which only 2 to biceps femoris muscle (Ali et al. 2011). Atypical presentations and unusual sites of metastasis from RCC create a diagnostic challenge in oncology.

We describe an unusual presentation of skeletal muscle metastasis from RCC and emphasize on the annual surveillance for metastatic RCC even after curative nephrectomy.

## Case

**Figure 1:**
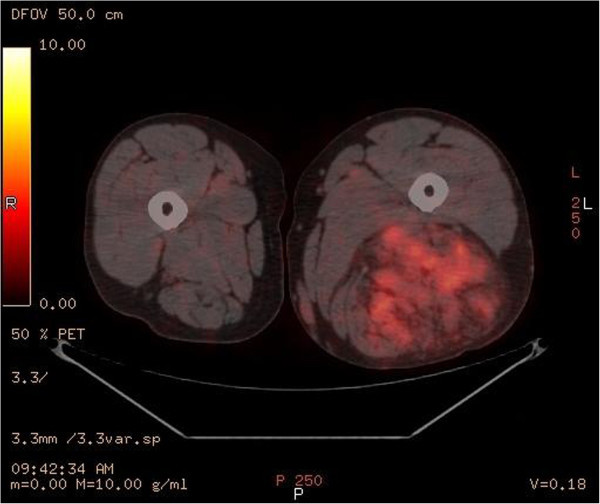
**Axial PET-IMG/CT shows hypermetabolic activity with standard uptake of 3.8 to 4.1 in left biceps femoris.**

**Figure 2:**
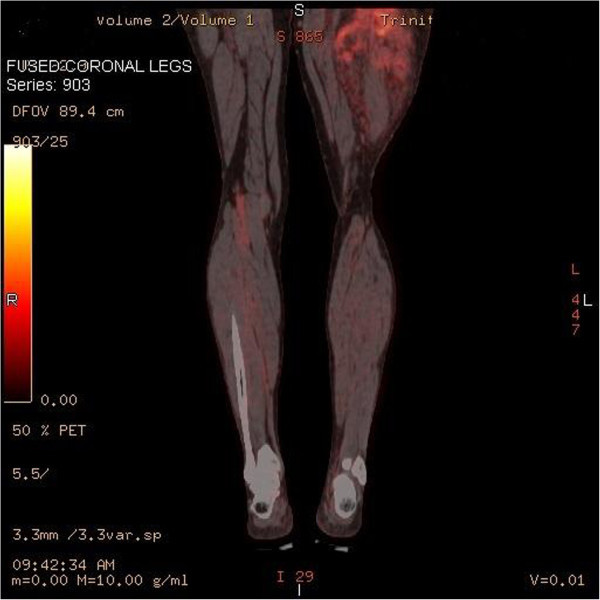
**A coronal PET-IMG/CT demonstrating significant enlargement of soft tissue and fatty components in the biceps femoris muscle belly.** Multiple serpiginous vessels are seen coursing through the soft tissue and surrounding edema.

**Figure 3:**
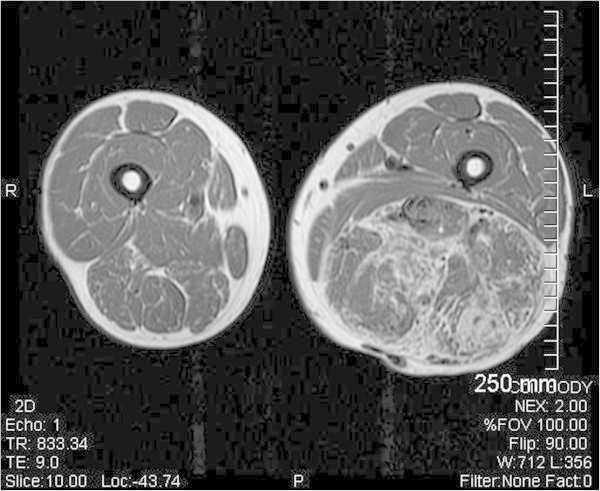
**T1-weighted axial MR image with fat saturation shows an encapsulated soft tissue mass with in the left biceps femoris muscle.**

**Figure 4:**
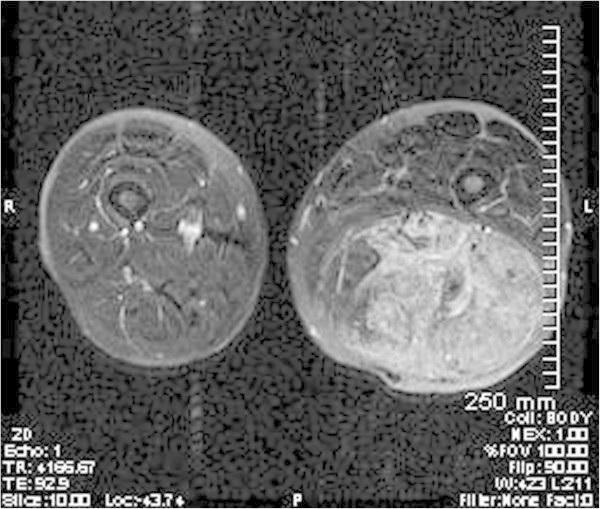
**A T2- weighted axial MR image showing avid enhancement of soft tissue components throughout the region indicating presence of various mesenchymal components including fat.** Multiple serpiginous enlarged draining and feeding vessels can also be identified.

**Figure 5:**
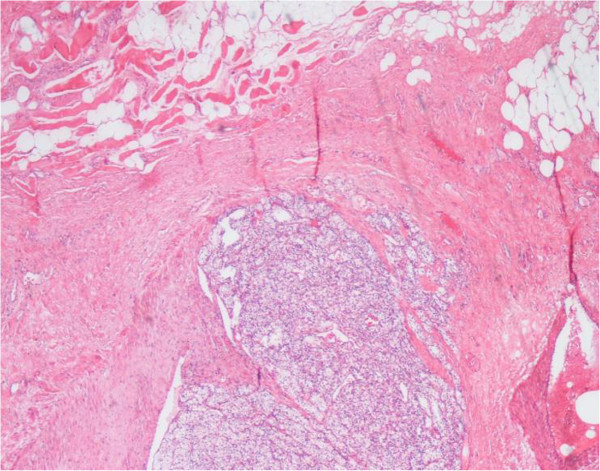
**Light microscopy 4X showing malignant cells with clear cytoplasm invading into surrounding soft tissue and skeletal muscle.**

**Figure 6:**
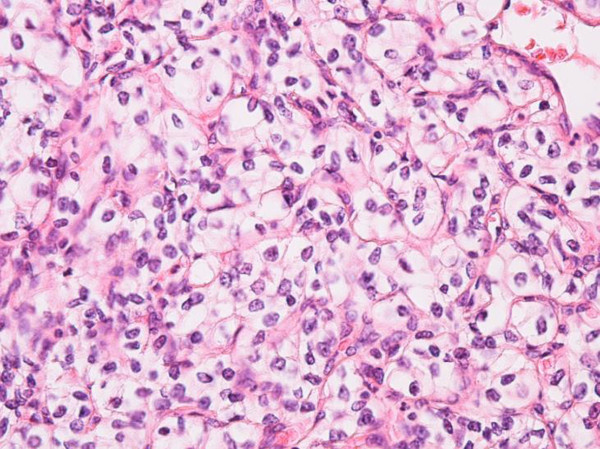
**Light microscopy 40X showing malignant cells with clear cytoplasm along with distinct cell membranes and prominent nucleoli.** It was positive for cytokeratin, RCC, p-NRA and negative for CK7, CK20, S-100, MART-1, and PAS.

We present a 58 year old male with an unusual posterior thigh mass for more than a year. Patient had a past medical history significant for RCC, initially diagnosed at stage II, 11 years ago followed by left nephrectomy. Patient also had metastasis to tail of the pancreas and tip of spleen 6 years ago which was followed by total resection of pancreas and spleen. Patient was followed up for RCC and was last seen 2 years ago when his PET/CT showed slightly increased hypermetabolic area in the biceps femoris muscle which was interpreted as a muscle injury secondary to the rarity of the metastasis to the skeletal muscle from RCC. Patient during the current follow up visit developed a large mass in the posterior side of the thigh which was present for more than a year. As per patient, the mass initially was diagnosed as a blood clot on venous doppler, for which he was treated by his primary care physician with warfarin for more than 6 months. However, the mass progressively increased in size. Patient did not mention of any other constitutional symptoms. On physical examination, a painless, tense mass along the length of biceps femoris muscle measuring more than 25 cm was found in the posterior aspect of the left thigh. The mass was hypervascular and numerous varicosities of different sizes were noticeable on the surface. Patient was imaged using FDG-PET/CT which showed hypermetabolic activity with an uptake value of 3.8 to 4.1 in biceps femoris muscle with multiple serpiginous vessels throughout the tumor, in accordance with a large cavernous hemangioma or an angiosarcoma. This was followed by MRI to better understand the morphology of the tumor, demonstrating a mesenchymal component in an encapsulated mass, raising the possibility of a liposarcoma or an angiosarcoma. A core tissue biopsy was done which demonstrated well defined adipose tissue but because of high suspicion for malignancy, patient underwent preembolization followed by surgical resection. A 28 x 17 x 7 cm resected mass was determined to be metastasis from his primary RCC (Figures 1, 2, 3, 4, 5 and 6).

Patient on his follow up with a whole body FDG-PET/CT was also found to have metastasis to his lateral ventricle in the brain from the RCC.

## Discussion

Renal cell carcinoma accounts for approximately 3% of all adult tumors (Sountoulides et al. 2011; Gözen et al. 2009) and about one third of cases present as metastasis either as initial presentation or late complication (Sountoulides et al. 2011; Gözen et al. 2009). Because of complex lymphatic drainage and early hematogenous spread (Gözen et al. 2009), atypical presentations and distant metastasis remain characteristic of RCC (Picchio et al. 2010; Ali et al. 2011; Schatteman et al. 2002; Sakamoto et al. 2007). Lung parenchyma, liver and bone are the common metastatic sites but it can also rarely spread to brain, pancreas, and skeletal muscle (Sountoulides et al. 2011; Pompo et al. 2008; Picchio et al. 2010; Hur et al. 2007; Taira et al. 2005; Gözen et al. 2009; Sakamoto et al. 2007).

It has been reported that approximately 0.4% of RCC metastasizes to skeletal muscle (Ali et al. 2011; Gözen et al. 2009; Sakamoto et al. 2007). Despite its rich blood supply and large surface area, skeletal muscle metastasis is very rare (Pompo et al. 2008; Pompo et al. 2009). Presence of protease inhibitors in the basement membrane inhibiting cell invasion, increased lactic acid levels and acidic environment interfering with tumor cell growth, presence of peptidic factors preventing the metastasis, muscular contractions dislodging the anchored tumor cells all provide insight into the rarity of skeletal metastasis. (Pompo et al. 2008; Taira et al. 2005; Ali et al. 2011; Gözen et al. 2009). Additionally, the scarcity or absence of specific receptors which influence the metastatic potential in RCC, the oncogene transgene bypassing the primary site and pre-selection of the organs of metastasis with formation of favorable environment by bone marrow cells may provide further explanation (Pompo et al. 2008).

RCC commonly metastasizes to soft tissues as a solitary soft tissue deposit developing any time between 6 months to 19 years, with the greatest risk in the first 5 years after initial presentation (Ali et al. 2011). McNichols et.al reported that only 11% of skeletal metastasis occurs after 10 years of primary tumor presentation (Hur et al. 2007; Camnasio et al. 2010; Sakamoto et al. 2007). The mass presented in this case, measuring 28 x 17 x 7 cm, is larger than previously reported sizes of these metastatic tumors, which have been in the range of 1.5 to 16 cm (Pompo et al. 2008).

Differentiating the unusual skeletal metastasis from other tumors still remains a diagnostic challenge and is pivotal because of the great differences in their treatment and prognosis (Pompo et al. 2008; Gözen et al. 2009). FDG-PET/CT, MRI helps in understanding the morphology of the tumor and a tissue biopsy is needed in most cases to make a definitive diagnosis (Hur et al. 2007; Sakamoto et al. 2007).

Location, size, and lack of pain in our patient were suspicious for diagnosis of soft tissue sarcomas (Pompo et al. 2008). Skeletal muscle metastasis as reported before presents as a painful mass and averages around 5.4 cm in size (Pompo et al. 2008). Presence of a hypervascular and large amount of varicosities was consistent with a hemangioma or angiosarcoma. Presence of the tumor in the skeletal belly is consistent with rhabdomyosarcoma. Though MRI and FDG-PET/CT could identify the tumor, they were not sufficient to make a definitive diagnosis. Tissue biopsy, known to have previously reported incorrect diagnoses in 13.5% of the cases in a study (Picchio et al. 2010), was negative in our case. The tumor mass on resection was differentiated on histopathology as a metastasis from clear cell RCC.

FDG-PET/CT is an effective tool in surveillance of RCC, known for its distant and unusual sites of metastasis (Picchio et al. 2010). It allows scanning the body in a single procedure combining functional information from PET which can be very useful in small sized tumors and anatomical details of CT (Picchio et al. 2010). Recent studies reported that FDG-PET/CT results are equivocal to any other modality in surveillance of metastasis in RCC (Picchio et al. 2010). To our knowledge no specific guidelines have been mentioned previously about surveillance in RCC after 10 years on an annual basis even for high grade tumors (>Pt2).

## Conclusion

We recommend maintaining a high level of suspicion for metastasis from RCC in a musculo-skeletal mass in a patient with RCC, even after curative nephrectomy. Because of advancement in treatment modalities, resulting in better survival rates, late presentation of the metastasis in RCC is increasing. We therefore recommend continuing intensive follow up for surveillance of the metastasis in RCC mainly in high grade tumors even after 10 years of initial presentation of the tumor or long disease free interval.

## Abbreviations

RCC: Renal cell carcinoma
SM: Skeletal muscle.
